# Risk factors for PTSD symptoms following PICU admission for childhood septic shock

**DOI:** 10.1007/s00787-024-02496-6

**Published:** 2024-06-15

**Authors:** Georgina J. Corbet Burcher, Lisa A. O’Dea, Mehrengise K. Cooper, Rebecca Lancaster, Robert A. McCutcheon, M. Elena Garralda, Simon Nadel

**Affiliations:** 1 Division of Psychiatry Imperial College, The Commonwealth Building, Du Cane Road, London, W12 0NN UK; 2 Department of Paediatric Intensive Care, St Mary’s Hospital, Imperial College Healthcare NHS Trust, London, UK; 3 Department of Psychiatry, University of Oxford, Oxford, UK

**Keywords:** PTSD, Inflammation, Sepsis, CRP, Psychopathology, Trauma

## Abstract

Objective

Paediatric intensive care unit (PICU) admission represents a traumatic event for many children. Follow-up studies have found post-traumatic stress disorder (PTSD) rates of 10–30%, with a particular prevalence following admission for sepsis. Dysregulated inflammatory responses are associated with PTSD. Sepsis involves a marked inflammatory response but the relationship between this and PTSD have not been clearly established. In this study we investigate associations between the inflammatory response, psychosocial risk factors, and PTS symptoms following PICU admission for septic shock.

We investigate the outcomes for children aged > 3 years, discharged from one PICU following admission for septic shock between 2010 and 2017. The study was a retrospective analysis of PICU-specific PTS symptoms reported by parents at any time since discharge via the Trauma and Behavior Health screen. Demographics, pre-morbid health characteristics, and exposure to other traumatic events were assessed. Clinical characteristics and blood test results at admission and at 48 h were recorded from clinical records. Multiple linear regression was used to investigate relationships between PTS symptom scores and predictor variables.

Data for 65 participants (48% male, median assessment age 8.0 years) was available. Median time since admission was 5.1 years. 30.8% children scored at risk of PTSD at any time since discharge Symptoms were significantly associated with acute CRP rise (p 0.03), other trauma exposures (p = 0.01), and female gender (p =0.04).

PTS symptoms in children who have survived septic shock are prevalent. These findings support a possible contribution of acute inflammatory changes, cumulative traumatic exposure, and female gender in post-PICU PTSD development.

**Supplementary Information:**

The online version contains supplementary material available at 10.1007/s00787-024-02496-6.

## Introduction

Critical illness requiring emergency admission to a paediatric intensive care unit (PICU) is a traumatic experience for many children and their families. Subsequent symptoms of post-traumatic stress disorder (PTSD) have been detected in 10–30% of children at 3–6 months following discharge [1, 2] and have been found to be particularly prevalent in those admitted with acute inflammatory disorders such as sepsis where prevalence rates for the disorder are estimated at around 20% [3]. PTSD is under-recognized in children and there is increasing acknowledgment that very young children can display PTSD features when exposed to traumatic events such as life-threatening illness. The UK prevalence of childhood PTSD is estimated to be 5–10% [4].

In the general population, PTSD in young people is more common in those with pre-existing psychological difficulties and/or low social support [4], those who have experienced multiple traumatic events and females [5]. Evidence regarding the effect of age at trauma on later symptom development is mixed [6]. The results from studies of PTSD in young survivors of critical illness are generally consistent with the above findings, however the small number of studies complicates generalisation of the conclusions [7].

In addition to these sociodemographic and environmental factors, it is possible that clinical and biological factors related to the acute illness may be significant in the development of PTSD in young people following a critical-illness related trauma. Longer length of PICU stay [1, 7], greater illness severity [8, 9] and use of midazolam [10] have been suggested to be associated with later development of PTS symptoms; and one study supports corticosteroids having a short-term protective effect [11]. In adult survivors of critical illness, the use of benzodiazepines and presentation of delirium are the strongest predictive factors for later development of PTS symptoms [12], but it is unknown if this is also the case in paediatric populations.

There is significant evidence to support associations with PTSD and inflammation, including increased levels of circulating pro-inflammatory cytokines [13, 14] and specific variabilities of the C-Reactive Protein (CRP) gene [15] in those diagnosed with the disorder. Furthermore, our previous exploratory work in children aged over 8 years with septic illness documented an association between PTS symptom burden at 3–6 months post PICU discharge and raised CRP levels in the first 48 h of admission [16].

In the current study we investigated a cohort of young people aged over 3 years at follow-up, admitted to one London (UK) PICU with septic shock. We retrospectively examined the presence of PTS symptoms specifically related to the PICU admission following discharge and associations with relevant sociodemographic and clinical risk factors. In addition, we investigated associations with the early acute inflammatory response. Our hypotheses were that PTS symptoms might be predicted by greater severity of PICU illness, greater inflammatory responses alongside pre-existing behavioural/emotional difficulties. We anticipated that use of steroids might be protective.

## Materials and methods

### Participant characteristics and recruitment

Participants were identified retrospectively from PICU records. Those admitted to PICU between 2010 and 2017 for over 24 h, who were diagnosed with septic shock based on clinical and laboratory assessment, and were over 3 years of age at follow-up were included. Exclusion criteria included prior or subsequent developmental disorder consistent with moderate/severe learning disability. Parents of participants who met inclusion criteria for the study were contacted via telephone or post to obtain informed consent.

### Power calculation

Due to a lack of pilot data, this study was powered to detect a clinically meaningful effect size. A power calculation demonstrated that a sample size of 60 would have 80% power to detect a correlation of r_p_ > 0.35 at an alpha of 0.05 [17].

### Collection of PICU clinical data

Data describing participants’ illness severity (Paediatric Index of Mortality 2 (PIM2) score [18], PICU length of stay, duration of intubation and ventilation), pharmacological interventions (use of corticosteroids, benzodiazepines, and opiates) were collected from medical records. Blood markers of an acute inflammatory response (CRP and total white cell count) were documented at the point of admission (T1) and at 48 h (T2). The change in value from T1 to T2 was calculated. CRP was chosen as an inflammatory indicator because of its clinical use in PICU as a measure of inflammation [19].

### Measures of demographics, physical and psychiatric morbidity

Parents of participants completed a questionnaire detailing recollection of the participants’ pre- and post-PICU overall status. Parents were asked to report on demographic, emotional and behavioural difficulties (determined as present by reports from school or health services), general health, and educational functioning.

PTS symptoms were evaluated using the Trauma and Behavioral Health Screen (TBH), a screening tool for use in community settings to detect risk of trauma-related sequelae in children > 3 years of age. It is an adaptation of the Young Child PTSD Checklist which has been shown to be a sensitive screen of PTS symptoms. The TBH has excellent face validity, covering the range of possible symptoms [20].

Two sections of the caregiver-report version were used in this study. Section 1 assessed for PTS symptoms *specifically relating to the PICU admission* (the specificity of symptoms related to the PICU admission and not to other traumatic events was stressed). Section 2 assessed for experiences and timing of other traumatic life events. Total scores on Section 1 varied from 0 to 45 with a total score > 10 indicating risk for clinical diagnosis of PTSD. Subscores were available for scoring of intrusion, avoidance, and hypervigilance symptoms. Parents were asked to report on PTS symptoms registered at their peak at any time since discharge.

### Statistical analysis

Statistical analyses were performed using the statistical programming language R (version 3.3.2 [21]). Statistical significance was set with a two-tailed *p* value of < 0.05.

An initial exploratory analysis employed bivariate linear regression to explore associations between potential risk factors and total PTS symptom scores. Investigated risk factors were as follows: demographic (age on admission to PICU, gender, parental status i.e. child living with two/one parent(s) or other, parental occupational status), clinical (PIM2, length of PICU stay, duration of intubation and ventilation, duration of use of benzodiazepines, opiates and use of corticosteroids), inflammatory (CRP change, white cell count change) and other (length of time since admission, other experience(s) of trauma and pre-PICU emotional or behavioural difficulties).

To assess for the combined effects of risk factors in determining PTS symptom scores, we constructed multiple variable linear regression models with relevant significant variables from the bivariate analyses used in this study alongside variables highlighted as possible predictors in previous similar studies.

## Results

The parents of 157 participants were approached and 65 (41%) returned the questionnaires. The median follow-up period between admission date and follow up date was 5.1 years.

Table 1 summarises the baseline demographic and overall health status of the cohort at the point of PICU admission. Median age on admission was 2 years and median age at follow-up was 8 years. There were similar numbers of males and females. Two thirds were of White British ethnicity; 65% were living with two parents. Twelve per cent (8/65) were reported by their parents as having pre-PICU emotional or behavioural difficulties.

**Table 1:** Demographic and pre-morbid general health characteristics of participants. Data are represented as median (interquartile range) or n/N (%)

Characteristic	Value
Age (years)	
• At PICU admission	2.0 (0.7–6.6)
• At study assessment	8.0 (5.6–11.7)
Gender	
• Male • Female	31/65 (48) 34/65 (52)
Parental Occupation status	
• NS-SEC Level I • NS-SEC Levels 2 & 3	24/65 (37) 34/65 (52)
Ethnicity	
• White Caucasian • Other	42/65 (66) 22/65 (34)
Family composition	
• Parental status (living with two parents) • Other	43/65 (65) 20/65 (31)
Pre-PICU psychological status	
• Prior emotional/behavioural difficulties	8/65 (12)
Other traumatic experiences	
• Total • Before PICU • After PICU • Timing unknown	21/65 (32) 9/21 (43) 7/21 (33) 5/21 (24)

NS-SEC National Statistics Socio-economic classification – stratification via parental occupational status, Level 1: Managerial, administrative, and professional occupations; Level 2: Intermediate Occupations; Level 3: Routine and manual occupations

Thirty-two per cent (21/65) of parents reported that their child had experienced other traumatic life events. In further detail, 24% (5/21) of these participants had been involved in a vehicle crash; 14% (3/21) had sustained life threatening injuries; 48% (10/21) designated the trauma category as ‘other’.

Participants’ clinical details including psychoactive medications used in PICU and inflammatory markers during the admission are reported in Table 2. The median length of PICU stay was 7 days. 37% received corticosteroids. Median CRP on admission (T1) was 100 mg/L and was 143.5 mg/L at T2 resulting in a median acute change of 11 mg/L.

**Table 2:** Clinical characteristics of participants during PICU admission. Data are represented as median (interquartile range) or n/N (%). T0 is at point of admission. T2 is at 48 h following admission. PIM2 (Paediatric Index of Mortality 2 Score)

Characteristic	Value
Length of PICU stay (days)	7.0 (5.0–11.0)
Length of intubation and ventilation (hours)	146 (79–216)
PIM2	10.90 (6.5-14.32)
**Pharmacological**	
Length of benzodiazepine use (hours)	120 (46–188)
Length of opiate use (hours)	138 (63–207)
Corticosteroid use	24/65 (37)
**Inflammatory Markers**	
CRP (mg/L)	
• T1 • T2 • Change from T1-T2	100 (41-249.5) 143.5 (62-260.5) 11 (-44.5-95.5)
Total white cell count (11.0 × 10^9^/L)	
• T1 • T2 • Change from T1-T2	11.3 (5.8–23.1) 11.4 (6.7-16.85) -0.4 (-4.7-3.7)

### PTS Symptom scores

Median total PTS symptom score at any time since discharge was 4, and 31% (20/65) scored at risk of a diagnosis of PTSD over this time period.

### Risk factors for PTS symptoms

Bivariate linear regression found significant associations between PTS symptoms and age (estimate = 0.56, SE = 0.2, *p* = 0.008); CRP change (estimate = 0.02, SE = 0.01, *p* = 0.04); experience of other traumatic event(s) (both pre and post PICU) (estimate = 5.36, SE = 2.00, *p* = 0.009); and benzodiazepine use (estimate = 0.02, SE = 0.01, *p* = 0.02). There was no significant association for use of corticosteroids (estimate =-2.2, SE = 2.11, *p* = 0.3).

Subsequently, a multiple variable model was created including significant variables alongside variables previously shown to be associated with PTS symptoms. The overall multiple variable model was significant (*p* = 0.01). PTS symptom scores were associated with CRP change (estimate = 0.02, SE = 0.01, *p* = 0.03), exposure to other traumatic events (estimate = 0.02, SE = 0.01, *p* = 0.01). and female gender (estimate = -4.18, SE = 1.95, *p* = 0.04). Older age of admission was not statistically significant (Estimate = 0.41, SE = 0.21, *p* = 0.05). Table 3 demonstrates these results in detail.

**Table 3:** Bivariate and multiple variable linear regression analysis for total PTS symptom score at any point since discharge and individual/grouped risk factors. The overall R2 for the multiple variable model was 0.32, with an adjusted R2 of 0.23 (*p* = 0.002), * *p* < 0.05 was considered significant

	Bivariate Models			Multiple Variable model		
	Estimate	Std. Error	p	Estimate	Std. Error	p
(Intercept)	-	-	-	4.68	1.93	0.01*
Age in PICU, years	0.56	0.20	0.008*	0.41	0.21	0.05
Male	− 3.25	2.02	0.11	− 4.18	1.95	0.04*
CRP change, mg/L	0.02	0.01	0.04*	0.02	0.01	0.03*
Other trauma experience (either pre or post PICU)	5.36	2.00	0.009*	5.93	2.20	0.01*
Length of benzodiazepine use, hours	0.02	0.01	0.02*	− 0.01	0.01	0.28
Pre-PICU emotional/behavioural difficulties	1.34	3.00	0.66	− 0.08	2.81	0.98
Length of PICU stay, days	0.06	0.04	0.18	0.11	0.07	0.13
Corticosteroid use	− 2.2	2.11	0.30	-1.1	1.97	0.57

### Post hoc PTS symptom subscore analysis

We conducted additional post hoc bivariate and multiple regression analyses for the main PTSD subgroup scores of intrusion, avoidance and hypervigilance symptoms. In the bivariate models, intrusion symptoms were associated with CRP change (estimate = 0.007, SE = 0.003, *p* = 0.046) and other experiences of trauma (estimate = 2.31, SE = 0.68, *p* = 0.001). Avoidance symptoms were associated with age on admission to PICU (estimate = 0.15, SE = 0.03, *p* = 4.22 × 10^− 5^); benzodiazepine use (estimate = 0.006, SE = 0.006, *p* = 1.39 × 10^− 7^); and PICU length of stay (estimate = 0.04, SE = 0.01, *p* = 5.93 × 10^− 6^). Supplementary tables 4a-c demonstrate these results in detail.

## Discussion

This study investigates PTSD outcomes and associated risk factors for children aged over 3 years previously admitted to PICU with septic shock. We found that approximately a third of children were deemed at risk. We have shown associations between an early inflammatory response in PICU and subsequent PTS symptomatology. We also demonstrate associations between PTS symptoms and the experience of other traumatic events and female gender.

Our results confirm high rates of PTS symptoms in children following septic illness as previously recorded [2, 3]. They are in keeping with our previous study showing an association between CRP rise and PTS symptoms over a shorter follow up period (3–6 months) and therefore support a possible role of acute inflammation in the development of PTS symptoms in children after PICU admission. They also add support to the hypothesis that inflammatory dysregulation might pre-date PTS symptoms as opposed to being a sequalae of the illness [22]. The mechanisms behind this association are speculative; the inflammatory environment present in septic shock is likely to be implicated in neurotoxic effects on the brain. The harmful direct effect of pro-inflammatory cytokines on the hippocampus has been shown in mouse models [23]. As PTS symptoms develop secondary to maladaptive memory-formation, it is possible that this neurotoxic environment may interfere with accurate encoding of memories in the hippocampus. Intrusion symptoms (more directly related to memory processing than other PTS symptoms) and not avoidance or hypervigilance symptoms showed some association with an acute CRP rise. This may reflect a possible underlying sensitivity of the cerebral regions connected to memory-based PTS symptoms to acute rises in inflammatory mediators (22). It must be couched however, that this finding was detected on post-hoc analysis. Furthermore, in contrast to our previous findings [11], we did not observe an association between corticosteroid use and reduced PTS symptoms. It is possible that any early protective effects of steroid use also recede over time.

The current findings add weight to the widening literature exploring cumulative adverse childhood experiences and later psychiatric dysfunction. The strongest association for PTS symptoms related to their PICU admission in our sample was for exposure to other traumatic event(s). About a third of children were reported to have been exposed, a comparable rate to the general population , and our results therefore indicate that cumulative experiences of trauma may promote PTS symptom burden. It is possible that the effects are mediated through neurobiological and/or inflammatory sensitization processes, with earlier traumatic experiences ‘priming’ the developing brain for a later pathological response to further traumatic events [24]. Our post hoc analysis suggests that this might promote the development of intrusion symptoms particularly. It is also possible that this is a result of a psychological sensitivity in the form of increasing recall bias for traumatic events [25].

The risk for PTSD in our sample was high when compared with expected population rates [4] indicating prolonged psychological vulnerability in this cohort and pointing to the importance of supporting families and attending to these problems as they develop, rather than simply waiting for them to resolve over time.

### Strengths and limitations

Despite several years interlude between PICU admission and follow up, we managed to achieve a relatively high response rate. With an awareness that observational studies are limited in their power to make causal inferences, in addressing links between inflammation and psychological stress reactions, a particular strength of the study is its longitudinal design showing inflammatory changes predated the development of PICU-related PTS symptoms. Research in this area is often correlational and raises issues around bidirectionality and direction of causality between PTSD and inflammation [22]. Furthermore, as early identification is known to improve outcome in children who are at high risk of developing PTSD [26], this study helps to add weight to the consideration of whether risk factors may be able to be used in the complex task of detecting which children should be monitored from a mental health perspective.

There are, however, several limitations to our study. The response rate was comparable to other PICU follow-up studies but presents a possibility for selection bias. This was a single clinical centre study which potentially limits its generalisability. Those children whose caregivers were more likely to have the facility to respond to the questionnaires, have a proficient understanding of English, and older age during admission were more highly represented. The number of children studied, while large for studies examining inflammation-PTSD associations following PICU admission, is still relatively small and precludes more complex modelling of the data.

Furthermore, the detection of PTS symptoms was assessed using a questionnaire and not by direct clinical interview, which may overestimate symptom levels when compared to clinical assessment. The use of parental responses (as opposed to direct child-report of symptoms) was chosen to standardise across the age range of participants but raises a limitation regarding measurement bias. The time delay also poses a risk of recall bias for children who were admitted a longer time ago. Some PTS symptoms may not also be specific to the PICU admission – particularly those representing hypervigilance. Psychosocial confounders were assessed for in a succinct manner and a larger more-detailed study (also including the effect of parental PTSD and psychological dysfunction) would be required to investigate these issues more thoroughly. Furthermore, we did not have a specific measure of delirium for the children and therefore were not able to add this into the regression models as a potential variable, however the use of benzodiazepines can be thought of as a useful proxy. The use of CRP as a marker for inflammation whilst helpful in a clinical sample, is a somewhat crude measure of inflammation more generally.

## Conclusions

Our study demonstrates novel findings regarding risk factors for PTS symptoms in children admitted to PICU with septic shock. The results show that children who experience a greater inflammatory response may be primed for post-traumatic stress symptomatology - particularly experiencing intrusion symptoms. Females and those who have been exposed to other prior traumatic events are more proneto PTS sympto. It is possible that very young children are less likely to be at risk. The findings have potential implications for both understanding the aetiology of post-PICU PTSD and for possible clinical interventions including screening for individuals at high risk of developing the disorder.

## Electronic supplementary material

Below is the link to the electronic supplementary material.

Supplementary Material 1

Supplementary Material 2

## Data Availability

No datasets were generated or analysed during the current study.

## References

[1] Als LC, Picouto MD, Hau S et al (2015) Mental and Physical Well-being following admission to Pediatric Intensive Care. 141–149. 10.1097/PCC.0000000000000424 10.1097/PCC.0000000000000424 25901544

[2] Ko MSM, Poh PF, Heng KYC et al (2022) Assessment of Long-Term Psychological outcomes after Pediatric Intensive Care Unit Admission: a systematic review and Meta-analysis. JAMA Pediatr 176:E215767. 10.1001/jamapediatrics.2021.5767 35040918 10.1001/jamapediatrics.2021.5767 PMC8767488

[3] Als LC, Picouto MD, O’Donnell KJ et al (2017) Stress hormones and posttraumatic stress symptoms following paediatric critical illness: an exploratory study. Eur Child Adolesc Psychiatry 26:511–519. 10.1007/s00787-016-0933-3 27995329 10.1007/s00787-016-0933-3 PMC5394132

[4] Lewis SJ, Arseneault L, Caspi A et al (2019) The epidemiology of trauma and post-traumatic stress disorder in a representative cohort of young people in England and Wales. Lancet Psychiatry 6:247–256. 10.1016/S2215-0366(19)30031-8 30798897 10.1016/S2215-0366(19)30031-8 PMC6384243

[5] Trickey D, Siddaway AP, Meiser-Stedman R et al (2012) A meta-analysis of risk factors for post-traumatic stress disorder in children and adolescents. Clin. Psychol. Rev 10.1016/j.cpr.2011.12.001 22245560

[6] Foy DW, Madvig BT, Pynoos RS, Camilleri AJ (1996) Etiologic factors in the development of posttraumatic stress disorder in children and adolescents. J Sch Psychol. 10.1016/0022-4405(96)00003-9

[7] Lopes-júnior LC, Antonia M, Rosa DP (2018) Rev Cohort Stud 19:16–21. 10.1097/PCC.0000000000001390

[8] Rees G, Gledhill J, Garralda ME, Nadel S (2004) Psychiatric outcome following paediatric intensive care unit (PICU) admission: a cohort study. Intensive Care Med 30:1607–1614. 10.1007/s00134-004-2310-9 15112035 10.1007/s00134-004-2310-9

[9] Rennick JE, Rashotte J (2009) Psychological outcomes in children following pediatric intensive care unit hospitalization: a systematic review of the research. J Child Heal Care 13:128–149. 10.1177/1367493509102472 10.1177/1367493509102472 19458168

[10] Long D, Gibbons K, Le Brocque R et al (2022) Midazolam exposure in the paediatric intensive care unit predicts acute post-traumatic stress symptoms in children. Aust Crit Care 35:408–414. 10.1016/j.aucc.2021.06.004 34373171 10.1016/j.aucc.2021.06.004

[11] Corbet Burcher G, Picouto MD, Als LC et al (2017) Post-traumatic stress after PICU and corticosteroid use. 10.1136/archdischild-2017-314157. Arch Dis Child archdischild-2017-314157 10.1136/archdischild-2017-314157 29175821

[12] Davydow DS, Gifford JM, Desai SV et al (2008) Posttraumatic stress disorder in general intensive care unit survivors: a systematic review. Gen Hosp Psychiatry 30:421–434. 10.1016/j.genhosppsych.2008.05.006 18774425 10.1016/j.genhosppsych.2008.05.006 PMC2572638

[13] Song H, Fang F, Arnberg FK et al (2019) Stress related disorders and risk of cardiovascular disease: Population based, sibling controlled cohort study. BMJ 365. 10.1136/bmj.l1255 10.1136/bmj.l1255 PMC6457109 30971390

[14] Pervanidou P (2008) Biology of post-traumatic stress disorder in Childhood and Adolescence. J Neuroendocrinol 20:632–638. 10.1111/j.1365-2826.2008.01701.x 18363804 10.1111/j.1365-2826.2008.01701.x

[15] Michopoulos V, Rothbaum AO, Jovanovic T et al (2015) CRP genetic variation and CRP levels are associated with increased PTSD symptoms and physiological responses in a highly traumatized civilian population. Am J Psychiatry 172:353–362. 10.1176/appi.ajp.2014.14020263 25827033 10.1176/appi.ajp.2014.14020263 PMC4440454

[16] Caspani G, Burcher GC, Garralda ME et al Inflammation and psychopathology in children following PICU admission: an exploratory study. 139–144 10.1136/ebmental-2018-300027 PMC6241628 30301824

[17] Erdfelder E, FAul F, Buchner A, Lang AG (2009) Statistical power analyses using G*Power 3.1: tests for correlation and regression analyses. Behav Res Methods 41:1149–1160. 10.3758/BRM.41.4.1149 19897823 10.3758/BRM.41.4.1149

[18] Lacroix J, Cotting J (2005) Severity of illness and organ dysfunction scoring in children. Pediatr Crit Care Med. 10.1097/01.PCC.0000161287.61028.D4. 6:15857545 10.1097/01.PCC.0000161287.61028.D4

[19] Póvoa P, Coelho L, Dal-Pizzol F et al (2023) How to use biomarkers of infection or sepsis at the bedside: guide to clinicians. Intensive Care Med 49:142–153. 10.1007/s00134-022-06956-y 36592205 10.1007/s00134-022-06956-y PMC9807102

[20] Mai TA, Scheeringa MS (2021) Caregiver and child agreement on traumatic events, PTSD, internalizing, externalizing, and ADHD problems in a child welfare population. J Public Child Welf 15:251–274. 10.1080/15548732.2019.1701612

[21] Team RCR A Language and Environment for Statistical Computing. https://www.r-project.org

[22] Sumner JA, Nishimi KM, Koenen KC et al (2020) Posttraumatic stress disorder and inflammation: untangling issues of Bidirectionality. 10.1016/j.biopsych.2019.11.005. Biol Psychiatry 6:10.1016/j.biopsych.2019.11.005 PMC7211139 31932029

[23] Wang Z, Young MRI (2016) PTSD, a disorder with an immunological component. Front Immunol 7:1–6. 10.3389/fimmu.2016.00219 27375619 10.3389/fimmu.2016.00219 PMC4893499

[24] Danese A, Baldwin JR (2017) Hidden wounds? Inflammatory links between Childhood Trauma and Psychopathology. Annu Rev Psychol 68:517–544. 10.1146/annurev-psych-010416-044208 27575032 10.1146/annurev-psych-010416-044208

[25] Shemesh E, Newcorn JH, Rockmore L et al (2005) Comparison of parent and child reports of emotional trauma symptoms in pediatric outpatient settings. Pediatrics 115. 10.1542/peds.2004-2201 10.1542/peds.2004-2201 15867023

[26] Haag AC, Landolt MA, Kenardy JA et al (2020) Preventive intervention for trauma reactions in young injured children: results of a multi-site randomised controlled trial. J Child Psychol Psychiatry Allied Discip 61:988–997. 10.1111/jcpp.13193 10.1111/jcpp.13193 31912485

