# Supplementary material for: Risk factors for PTSD symptoms following PICU admission for childhood septic shock
Source: Eur Child Adolesc Psychiatry. 2024 Jun 15;34(1):307–13. doi: 10.1007/s00787-024-02496-6 (PMC11805800; doi:10.1007/s00787-024-02496-6)
Supplement: Supplementary file 1 — Supplementary Material 1 [file 787_2024_2496_MOESM1_ESM.docx]

**Stress symptoms following admission to PICU**

**and effects of medication**

**Parent/Guardian TBH Questionnaire**

Date

Name of child

Your name

Relationship to child

Age of child

**PART ONE**

- Below is a list of problems that children sometimes have after being admitted to intensive care with a serious condition.
- Read each one carefully and circle the number (0-3) that best describes how often that problem has bothered your child. Rate the symptoms when they were at their worst.
- The questions being asked refer **to the events of your child’s admission to intensive care.**

1. = Not at all/only once
2. = Once a week or less/once in a while

2 = 2- 4 times a week/half the time

3 = 5 or more times a week/almost always

| 1. Having upsetting thoughts or images about his/her time in hospital that came into my child’s head when he/she didn’t want them to |  | 0 | 1 | 2 | 3 |
| --- | --- | --- | --- | --- | --- |
| 1. Having bad dreams or nightmares |  | 0 | 1 | 2 | 3 |
| 1. Acting or feeling as if it was happening again (e.g. hearing something or seeing a picture about it, and feeling as if he/she is there again) |  | 0 | 1 | 2 | 3 |
| 1. Feeling upset when he/she thinks or hears about it (feeling scared, angry, sad, guilty, etc.) |  | 0 | 1 | 2 | 3 |
| 1. Having feelings in his/her body when thinking about or hearing about it (e.g. breaking out into a sweat, heart beating fast) |  | 0 | 1 | 2 | 3 |
| 1. Trying not think about, talk about or have feelings about it. |  | 0 | 1 | 2 | 3 |
| 1. Trying to avoid activities, people, or places that remind him/her of it. |  | 0 | 1 | 2 | 3 |
| 1. Having much less interest in doing things he/she used to do |  | 0 | 1 | 2 | 3 |
| 1. Not feeling close to people around him/her |  | 0 | 1 | 2 | 3 |
| 1. Not being able to have strong feelings (e.g. being unable to cry or unable to feel happy) |  | 0 | 1 | 2 | 3 |
| 1. Feeling as if his/her future plans or hopes will not come true (e.g., feeling like he/she will not have a job, get married, or have kids) |  | 0 | 1 | 2 | 3 |
| 1. Having trouble falling or staying asleep |  | 0 | 1 | 2 | 3 |
| 1. Feeling irritable or having fits of anger |  | 0 | 1 | 2 | 3 |
| 1. Being overly careful (e.g. checking who and what is around) |  | 0 | 1 | 2 | 3 |
| 1. Being jumpy or easily startled   (e.g. jumping when someone walks up behind him/her) |  | 0 | 1 | 2 | 3 |

**PART TWO**

Has your child ever been involved in any of the following traumatic events?

To count an event he/she must have:

- (1)  Felt like he/she might die OR
- (2)  He/she had a serious injury or felt like he/she might get a serious injury, OR
- (3) He/she felt extremely upset by
- (4)  He/she saw (1) or (2) happen to another person, or saw someone die.

|  | **Did this event happen?** | | | **Age (the last time this happened)** | | | **Age (the first time this happened)** | | | **How many times**  **did this happen?** | | | |
| --- | --- | --- | --- | --- | --- | --- | --- | --- | --- | --- | --- | --- | --- |
| 1. **Crash in car/plane/**   **Boat** | No | Yes | Not sure | 0-6 | 7-12 | 13-18 | 0-6 | 7-12 | 13-18 | 1 | 2-5 | 6-9 | >10 |
| 1. **Attacked by an animal** | No | Yes | Not sure | 0-6 | 7-12 | 13-18 | 0-6 | 7-12 | 13-18 | 1 | 2-5 | 6-9 | >10 |
| 1. **Natural disaster**   **(flood, hurricane, war, fire)** | No | Yes | Not sure | 0-6 | 7-12 | 13-18 | 0-6 | 7-12 | 13-18 | 1 | 2-5 | 6-9 | >10 |
| 1. **Physical abuse** | No | Yes | Not sure | 0-6 | 7-12 | 13-18 | 0-6 | 7-12 | 13-18 | 1 | 2-5 | 6-9 | >10 |
| 1. **Sexual abuse/sexual assault/rape** | No | Yes | Not sure | 0-6 | 7-12 | 13-18 | 0-6 | 7-12 | 13-18 | 1 | 2-5 | 6-9 | >10 |
| 1. **Life-threatening injuries** | No | Yes | Not sure | 0-6 | 7-12 | 13-18 | 0-6 | 7-12 | 13-18 | 1 | 2-5 | 6-9 | >10 |
| 1. **Witnessed another person being beaten/raped/threatened with serious harm/killed** | No | Yes | Not sure | 0-6 | 7-12 | 13-18 | 0-6 | 7-12 | 13-18 | 1 | 2-5 | 6-9 | >10 |
| 1. **Other traumatic event** | No | Yes | Not sure | 0-6 | 7-12 | 13-18 | 0-6 | 7-12 | 13-18 | 1 | 2-5 | 6-9 | >10 |

1. If more than one event happened to your child indicate the one which caused most distress to him/her:
2. Did these events happen before or after admission to PICU?
