# Supplementary material for: Risk factors for PTSD symptoms following PICU admission for childhood septic shock
Source: Eur Child Adolesc Psychiatry. 2024 Jun 15;34(1):307–13. doi: 10.1007/s00787-024-02496-6 (PMC11805800; doi:10.1007/s00787-024-02496-6)
Supplement: Supplementary file 2 — Supplementary Material 2 [file 787_2024_2496_MOESM2_ESM.docx]

Supplementary Table 4a & 4b & 4c

Bivariate and multiple variable regression analysis for intrusion/avoidance symptom subscores at any time since discharge and individual risk factors

1. Intrusion symptom subscore. Overall multiple variable regression model had R^2^ of 0.31 with adjusted R^2^ of 0.21 (p=0.008), * p<0.05 was considered significant.

|  | **Bivariate Models** | | | **Multiple Variable Model** | | |
| --- | --- | --- | --- | --- | --- | --- |
|  | Estimate | Std. Error | p | Estimate | Std. Error | p |
| (Intercept) | - | - | - | 1.84 | 0.69 | 0.01* |
| Age in PICU, *years* | 0.14 | 0.08 | 0.07 | 0.10 | 0.07 | 0.17 |
| Male | -1.16 | -0.75 | 0.12 | -1.55 | 0.69 | 0.03* |
| CRP change, *mg/L* | 0.007 | 0.003 | 0.046* | 0.005 | 0.003 | 0.11 |
| Other trauma experience | 2.31 | 0.68 | 0.001* | 2.47 | 0.78 | 0.003* |
| Length of benzodiazepine use, *hours* | 0.005 | 0.002 | 0.06 | -0.004 | 0.004 | 0.43 |
| Pre-PICU emotional/behavioural difficulties | 0.09 | 1.05 | 0.93 | -0.30 | 1.00 | 0.77 |
| Length of stay, *days* | 0.004 | 0.02 | 0.82 | 0.016 | 0.03 | 0.56 |
| Corticosteroid use | -0.94 | 0.78 | 0.24 | -0.25 | 0.70 | 0.72 |

1. Avoidance symptom subscore. Overall multiple variable regression model had R^2^ of 0.51 with adjusted R^2^ of 0.44 (p=2x10^-6^), * p<0.05 was considered significant.

|  | **Bivariate Models** | | | **Multiple Variable Model** | | |
| --- | --- | --- | --- | --- | --- | --- |
|  | Estimate | Std. Error | p | Estimate | Std. Error | p |
| (Intercept) | - | - | - | -0.21 | 0.31 | 0.51 |
| Age in PICU, years | 0.15 | 0.03 | 4.22x10^-5^* | 0.11 | 0.03 | 0.002* |
| Male | -0.26 | 0.38 | 0.49 | -0.51 | 0.31 | 0.11 |
| CRP change, mg/L | 0.002 | 0.002 | 0.26 | 0.002 | 0.001 | 0.22 |
| Other trauma experience | 0.58 | 0.38 | 0.13 | 0.26 | 0.35 | 0.46 |
| Length of benzodiazepine use, hours | 0.006 | 0.001 | 1.39x10^-7^ * | 0.002 | 0.002 | 0.33 |
| Pre-PICU emotional/behavioural difficulties | 0.43 | 0.55 | 0.44 | 0.17 | 0.45 | 0.71 |
| Length of stay, days | 0.04 | 0.01 | 5.93x10^-6^* | 0.02 | 0.01 | 0.10 |
| Corticosteroid use | 0.09 | 0.40 | 0.81 | 0.27 | 0.31 | 0.39 |

1. Hypervigilance symptom subscore. Overall multiple variable regression model had R^2^ of 0.16 with adjusted R^2^ of 0.029 (p=0.3), * p<0.05 was considered significant.

|  | **Bivariate Models** | | | **Multiple Variable Model** | | |
| --- | --- | --- | --- | --- | --- | --- |
|  | Estimate | Std. Error | p | Estimate | Std. Error | p |
| (Intercept) | - | - | - | 4.03 | 1.08 | 0.0005* |
| Age in PICU, years | 0.11 | 0.1 | 0.29 | 0.08 | 0.11 | 0.48 |
| Male | -1.67 | 0.95 | 0.08 | -1.8 | 1.08 | 0.09 |
| CRP change, mg/L | 0.005 | 0.004 | 0.25 | 0.007 | 0.005 | 0.15 |
| Other trauma experience | 1.88 | 1.01 | 0.07 | 2.19 | 1.22 | 0.08 |
| Length of benzodiazepine use, hours | 0.002 | 0.003 | 0.55 | -0.005 | 0.007 | 0.46 |
| Pre-PICU psychiatric problem | 0.032 | 1.48 | 0.83 | -0.09 | 1.56 | 0.95 |
| Length of stay, days | -0.003 | 0.02 | 0.88 | 0.02 | 0.04 | 0.58 |
| Corticosteroid use | -0.078 | 0.98 | 0.44 | -0.43 | 1.09 | 0.69 |
